# Next‐Generation Sequencing for Characterizing Respiratory Tract Virome and Improving Detection of Viral Pathogens in Children With Pneumonia

**DOI:** 10.1111/irv.13362

**Published:** 2024-08-09

**Authors:** Song Cui, Ruochun Guo, Changming Chen, Yue Zhang, Jinxin Meng, Lanxin Liu, Yanxia Li, Zhijie Kang, Shenghui Li, Qiulong Yan, Yufang Ma

**Affiliations:** ^1^ Department of Biochemistry and Molecular Biology, College of Basic Sciences Dalian Medical University Dalian China; ^2^ Department of Critical Care Medicine Dalian Municipal Central Hospital Dalian China; ^3^ Puensum Genetech Institute Wuhan China; ^4^ Department of Rheumatology and Immunology The Second Affiliated Hospital of Guizhou University of Traditional Chinese Medicine Guiyang China; ^5^ Department of Hematology The Second Hospital of Dalian Medical University Dalian China; ^6^ Department of Microbiology, College of Basic Sciences Dalian Medical University Dalian China

**Keywords:** child pneumonia, pathogen, pneumonia, respiratory virome, virome

## Abstract

**Background:**

Pneumonia is typically caused by a variety of pathogenic microorganisms. Traditional research often focuses on the infection of a few microorganisms, whereas metagenomic studies focus on the impact of the bacteriome and mycobiome on respiratory diseases. Reports on the virome characteristics of pediatric pneumonia remain relatively scarce.

**Methods:**

We employed de novo assembly and combined homology‐ and feature‐based methods to characterize the respiratory virome in whole‐genome DNA sequencing samples from oropharynx (OP) swabs, nasopharynx (NP) swabs, and bronchoalveolar lavage fluids (BALF) of children with pneumonia.

**Results:**

Significant differences were observed in the alpha and beta diversity indexes, as well as in the composition of the oropharyngeal virome, between pneumonia cases and controls. We identified 1137 viral operational taxonomic units (vOTUs) with significant differences, indicating a preference of pneumonia‐reduced vOTUs for infecting *Prevotella*, *Neisseria*, and *Veillonella*, whereas pneumonia‐enriched vOTUs included polyomavirus, human adenovirus, and phages targeting *Staphylococcus*, *Streptococcus*, *Granulicatella*, and *Actinomyces*. Comparative analysis revealed higher relative abundances and prevalence rates of pneumonia‐enriched OP vOTUs in NP and BALF samples compared to pneumonia‐reduced vOTUs. Additionally, virome analysis identified six pediatric patients with severe human adenovirus or polyomavirus infections, five of whom might have been undetected by targeted polymerase chain reaction (PCR)‐based testing.

**Conclusions:**

This study offers insights into pediatric pneumonia respiratory viromes, highlighting frequent transmission of potentially pathogenic viruses and demonstrating virome analysis as a valuable adjunct for pathogen detection.

## 1 Introduction

Pneumonia, which is lung inflammation caused by pathogens like bacteria and viruses, as well as other factors such as the inhalation of amniotic fluid or meconium, has shown declining mortality and incidence rates year after year, even following the COVID‐19 outbreak [1, 2, 3]. Nonetheless, it remains the most common respiratory disease in childhood and the leading cause of death in children under 5 years old [2, 4]. This has led to widespread attention on the microbial ecosystem of the respiratory tract, making the detection of respiratory microbes one of the most common applications of next‐generation sequencing (NGS) in practical use.

Usually, lung microorganisms are derived from those colonizing the oropharynx and upper respiratory tract (URT). NGS has broadened the horizons of respiratory tract microbiota research by allowing for a thorough investigation of both known and previously unidentified microbial communities, rather than being confined to the study of a limited number of known microbes. Current researches on the microbiome of the respiratory tract have mainly focused on bacteria and fungi. The normal respiratory tract microbiota prevent pneumonia by preventing the colonization of potentially pathogenic bacteria and modulating the immune response. However, when the abovementioned microbiota is disturbed or microbe–host crosstalk occurs, respiratory tract and lung functions are compromised and susceptible to pneumonia [5, 6, 7]. This disruption of homeostasis often accompanies the outbreak of infections by pathogens in the respiratory system, including bacteria like *Streptococcus pneumoniae* , *Haemophilus influenzae* , and *Klebsiella pneumoniae* , as well as the fungus *Candida albicans* [8, 9].

Viruses as the key agents in many respiratory diseases, especially pneumonia, interact with the human immune system. However, previous technical obstacles, such as the absence of conserved nucleic acid sequences in viruses and the difficulty of viral isolation and purification, have limited access to more virus‐related information. Therefore, most studies targeting respiratory tract viruses use targeted detection of the limited number of known viruses, revealing various pathogens responsible for pneumonia, such as influenza virus, respiratory syncytial virus, rhinovirus, and coronavirus [10, 11, 12]. These studies often address questions in virology without considering ecological aspects. In recent years, metagenomics‐based virome studies have uncovered a huge amount of unexplored viral taxa in various human body sites, including the gut, oral cavity, skin, and reproductive tract [13, 14, 15, 16, 17], which underscores a more complex viral ecology than previously recognized. Previous studies have shown that the respiratory virome regulates the host immunity since childhood and the human lungs are filled with disease‐unrelated viruses [18]. The increased respiratory viral load or suppression of host antiviral capacity may affect host physiology and cause chronic lung diseases, including asthma, chronic obstructive pulmonary disease (COPD), cystic fibrosis, or acute lung diseases, such as COVID‐19, which may lead to the pathogenesis of these diseases [19, 20, 21]. These findings provide insight into the importance of an ecological perspective for understanding the changes of diversity and composition in the respiratory tract virome during pediatric pneumonia, which is crucial for exploring the potential mechanisms of disease pathogenesis.

To identify changes in the respiratory tract virome in children with pneumonia, we reanalyzed the public respiratory tract metagenomes sequenced from the oropharynx (OP) swab (*n* = 75), nasopharynx (NP) swab (*n* = 42), and bronchoalveolar lavage fluid (BALF; *n* = 46) specimens of 76 child patients with pneumonia and the OP specimens (*n* = 171) of 171 healthy children. These metagenomic samples were originally used to explore the bacteriome characteristics of pediatric pneumonia populations [22]. Here, we compared the viral composition of pediatric pneumonia patients and healthy children using de novo assembly and the combined homology‐ and feature‐based methods for viral identification to comprehensively characterize respiratory tract virome composition in whole‐genome DNA sequencing samples derived from OP swabs, NP swabs, and BALF of children with pneumonia. A better understanding of the etiology and pathogenesis of pediatric pneumonia based on respiratory tract virome exploration will contribute to the development of new prevention and a timely and auxiliary diagnosis of pathogen detection.

## 2 Methods

### 2.1 Data Collection and Preprocessing

All 334 respiratory tract metagenomes in this study come from Dai et al.'s study and are available under NCBI BioProject accession number PRJNA413615 [22]. These metagenomes sequenced from the OP swab (*n* = 75), NP swab (*n* = 42), and BALF (*n* = 46) specimens of 76 child patients with pneumonia and the OP specimens (*n* = 171) of 171 healthy children [22]. All OP and NP specimens were collected within 24 h of hospitalization and before antibiotic treatment, whereas BALF specimens were obtained during Days 2–15 after hospitalization [22]. Among 76 child patients with pneumonia, 39 were diagnosed with *Mycoplasma pneumoniae* pneumonia, 15 with microbial coinfected pneumonia, 12 with unexplained pneumonia, 3 with *S. pneumoniae* pneumonia, 3 with virus pneumonia caused by *respiratory syncytial virus* or *Epstein–Barr virus*, and 4 with other types of pneumonia. More metadata of subjects are presented in Table S1, including gender, age, disease status, delivery mode, and feed mode. The DNA extraction from the specimens was performed using the TGuide S32 Magnetic Swab Genomic DNA Kit (DP603‐T2, TIANGEN Biotech [Beijing] Co., Ltd., Beijing, China), and the sequencing was conducted on the Illumina HiSeq platform.

Raw metagenomic data were quality‐filtered by fastp v0.20.1 with the options “‐u 30 ‐q 20 ‐l 90 ‐y ‐‐trim_poly_g” [23]. In order to remove host contamination, the filtered reads were further mapped against the human genome GRCh38 via bowtie2 v2.4.1 [24]. The remaining reads of each sample were considered as the high‐quality non‐human reads (1.04 ± 0.92 Gb per sample). These reads were assembled into contigs by MEGAHIT v1.2.9 with the options “‐‐k‐list 21,41,61,81” [25], and only 409,845 assembled contigs with a sequence length of greater than 5000 bp were retained for the following viral identification.

The public oral virome database (OVD) was downloaded from https://github.com/RChGO/OVD [15]. The public oral prokaryotic genome database was downloaded from https://ftp.cngb.org/pub/SciRAID/Microbiome/human_oral_genomes/bowtie2_index [26].

### 2.2 Viral Sequence Processing

The viral sequence processing pipeline was conducted following our previous study [15]. In brief, we employed a de novo approach to detect candidate viral sequences using three tools: CheckV v0.7.0 [27], DeepVirFinder v1.0 [28], and VIBRANT v1.2.1 [29]. Candidate sequences with a bacterial universal single‐copy ortholog (BUSCO) ratio of ≥ 5% were considered as highly contaminated and thus filtered out [15]. Furthermore, we assessed the genome quality of the remaining sequences using CheckV v0.7.0. A total of 3097 viral sequences with a CheckV completeness of > 50% were retained and clustered into 2402 viral operational taxonomic units (vOTUs) at a 95% nucleotide identity threshold across 75% of the sequence. Taxonomic classification of vOTUs was performed by aligning protein sequences to a customized database [15]. Virus‐host prediction was carried out using two bioinformatic methods that included CRISPR‐spacer matches and prophage blasts [15]. For functional annotation, viral proteins were predicted using Prodigal v2.6.3 and annotated based on the Kyoto Encyclopedia of Genes and Genomes (KEGG) database using DIAMOND [30, 31, 32].

### 2.3 The Virome Composition of Respiratory Tract

To more comprehensively describe the virome composition of the respiratory tract, we clustered 2402 respiratory tract vOTUs and 21,090 OVD viruses with > 50% CheckV completeness at > 95% nucleotide similarity across > 75% of the sequence, generating a new viral genome catalog of 22,860 vOTUs. The addition of viruses from the OVD was motivated by the similarity in sample types, aiming to enhance the diversity and completeness of our viral genomes by leveraging this large and comparable viral metagenomic database. The high‐quality nonhuman reads of each metagenome were mapped against this viral genome catalog by bowtie2 v2.4.1 [24] with the options “‐‐end‐to‐end ‐‐fast ‐‐no‐unal.” Based on the mapped read counts, we calculated the relative abundances of vOTUs in each sample. Specifically, for a vOTU i, if $N_{i}$ reads mapped against it in a sample, we calculated the average base depth $S_{i}$ of vOTU i using the formula $S_{i}=\frac{N_{i}}{L_{i}}$. Here, $L_{i}$ represents the genome size of vOTU i. Then, the relative abundance $R_{i}$ of vOTU i in the sample was determined by dividing its base depth $S_{i}$ by the sum of base depths of all vOTUs in the sample: $R_{i}=\frac{S_{i}}{\sum_{j\in \mathrm{all}}S_{j}}$. In term of each viral family, its relative abundance was calculated by summing the relative abundances of the vOTUs associated with this family.

### 2.4 Availability of Data

All respiratory tract metagenomes data in this study are available under NCBI BioProject accession number PRJNA413615. The public OVD was downloaded from https://github.com/RChGO/OVD [15]. The public oral prokaryotic genome database was downloaded from https://ftp.cngb.org/pub/SciRAID/Microbiome/human_oral_genomes/bowtie2_index [26].

### 2.5 Statistical Analyses

Statistical analyses and visualizations were carried out in R v4.0.3.

#### 2.5.1 Alpha Diversity

The number of observed vOTUs in each sample was calculated as the count of vOTUs with the nonzero relative abundance. The rarefaction curve of the number of observed vOTUs was constructed by 10 times random subsampling of samples from 1 to maximum sample size in the step of 1. The Shannon index and Simpson index were estimated using the function *diversity* based on the vOTU‐level relative abundance profile.

#### 2.5.2 Beta Diversity

Inter‐sample Bray–Curtis distances were calculated using the function *vegdist*. Principal coordinate analysis (PCoA) was performed based on the Bray–Curtis distance based on the vOTU‐level abundance data using the function *pcoa* in the ape package. Permutational multivariate analysis of variance (PERMANOVA) was performed using the function *adonis*.

#### 2.5.3 Statistical Tests

We employed the Wilcoxon rank‐sum tests to determine the vOTUs that were differentially abundant between healthy controls (HC) and pneumonia patients (PP) by utilizing the function *wilcox.test*. *p* values were adjusted by the function *p.adjust* with the option “method = BH,” and vOTUs with the adjusted *p* value (*q* value) < 0.01 were positive. In order to reveal the functional differences between HC‐ and PP‐enriched vOTUs, we assessed the occurrence rate of each KEGG functional ortholog (KO) in the two groups. The occurrence rate of a KO in each group refers to the number of vOTUs with this KO divided by total number of vOTUs in that group. To perform statistical tests, we used Fisher's exact test with a 2 × 2 contingency table considering the presence or absence of a given KO in each group. A *p* value less than 0.01 was considered statistically significant.

#### 2.5.4 Classification Model

We applied random forest models with 10 repeats of 10‐fold cross‐validation to assess the diagnostic potential of oropharynx pneumonia‐associated vOTUs for the separation of patients with pneumonia from healthy controls using the function *randomForest*, and the model performance was estimated based on the area under the receiver operating characteristic curve (AUC) using the function *roc* in the pROC package. Briefly, in each repetition, all OP samples were randomly grouped into 10 equal folds. Then every fold in turn was designated as the testing set and the remaining nine folds as the training set of the model. The model performance was defined as the average AUC score generated from 10 repeats of 10‐fold cross‐validation.

## 3 Results

### 3.1 Study Population and the Virome Construction of the Human Respiratory Tract

**FIGURE 1:**
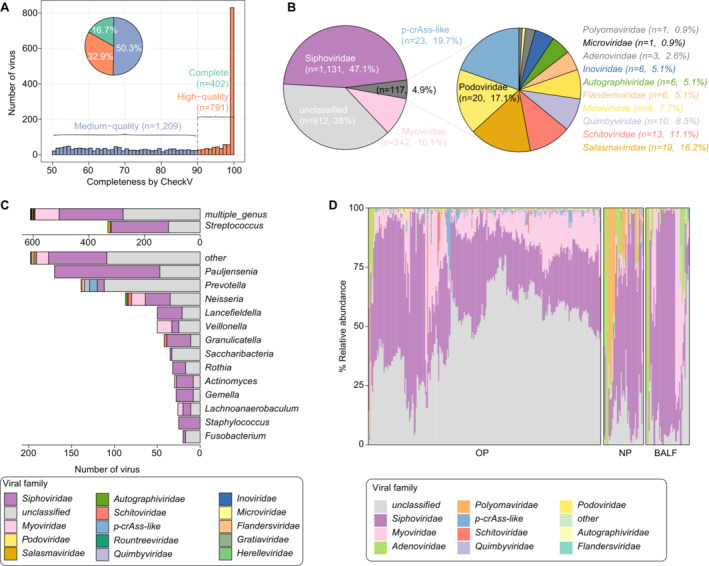
Overview of respiratory tract virome. (A) The count of vOTUs with the same genome quality evaluated by CheckV. (B) The count of vOTUs with the same viral family‐level annotation. (C) Bar plot showing the numbers of vOTUs infecting different prokaryotic hosts. The prokaryotic hosts are grouped at the genus level, and the viruses are colored based on their family‐level annotation. (D) The viral family‐level composition of respiratory tract samples. BALF, bronchoalveolar lavage fluids; NP, nasopharynx swabs; OP, oropharynx swabs.

This study reanalyzed the public respiratory tract metagenomes sequenced from the OP swab (*n* = 75), NP swab (*n* = 42), and BALF (*n* = 46) specimens of 76 child patients with pneumonia and the OP specimens (*n* = 171) of 171 healthy children [22]. We used de novo assembly alongside the combined homology‐ and feature‐based methods to identify virus sequences in each metagenome, resulting in a collection of a total of 3097 credible viral sequences with completeness > 50%. These viral sequences were further clustered into 2402 vOTUs based on 95% nucleotide similarity. The CheckV assessment [27] defined 16.7% vOTUs as complete, 32.9% as high‐quality, and 50.3% as medium‐quality genomes (Figure 1A and Table S2). The majority of 2402 vOTUs (90.8%) were annotated as DNA viruses, which might be due to that the viruses were recovered from whole‐metagenome DNA sequencing samples. Family‐level taxonomic annotation further showed that 62.0% vOTUs (*n* = 1490) could be assigned to known viral families (Figure 1B and Table S2). The majority of the annotated vOTUs (*n* = 1477) were belonging to families of prokaryotic viruses, such families as Siphoviridae (*n* = 1131), Myoviridae (*n* = 242), crAss‐like (*n* = 23), Podoviridae (*n* = 20) and Salasmaviridae (*n* = 19). Only 13 vOTUs were assigned to three families of eukaryotic DNA viruses, including nine Metaviridae, three Adenoviridae, and one Polyomaviridae members. Notably, the three Adenoviridae vOTUs showed 99.97%, 99.47%, and 99.95% nucleotide sequence similarity to *human adenovirus B3*, *human adenovirus C*, and *human adenovirus E4*, respectively, whereas the Polyomaviridae vOTU was identified as *WU polyomavirus* (100% nucleotide sequence similarity). Virus‐host prediction was performed using genomic alignments or matching of CRISPR spacers based on a large oral prokaryotic genome dataset of 3589 species [26], leading to 1891 vOTUs (78.7%) with at least one predicted host (Table S2). The prevalent hosts of these vOTUs were members of *Streptococcus*, *Pauljensenia*, *Prevotella*, *Neisseria*, *Lancefieldella*, and *Veillonella* (Figure 1C).

To more comprehensively characterize the virome of the respiratory tract, we integrated our vOTUs with viruses sourced from a large‐scale oral virome database (OVD), yielding a new viral genome catalog of 22,860 vOTUs (Table S3). The viral community composition of all respiratory tract metagenomes was profiled based on this new viral genome catalog. There was a remarkable difference in viral composition at the family level among OP, NP, and BALF metagenomes (Figure 1D). The OP samples were dominated by the family‐level unclassified viruses (50%), Siphoviridae (34%), and Myoviridae (12%). Besides these three families of prokaryotic viruses, NP and BALF metagenomes had a high proportion of Adenoviridae (15% and 2%) and Polyomaviridae (14% and 11%).

### 3.2 Case–Control Comparison of Diversity and Structure in Oropharynx Virome

**FIGURE 2:**
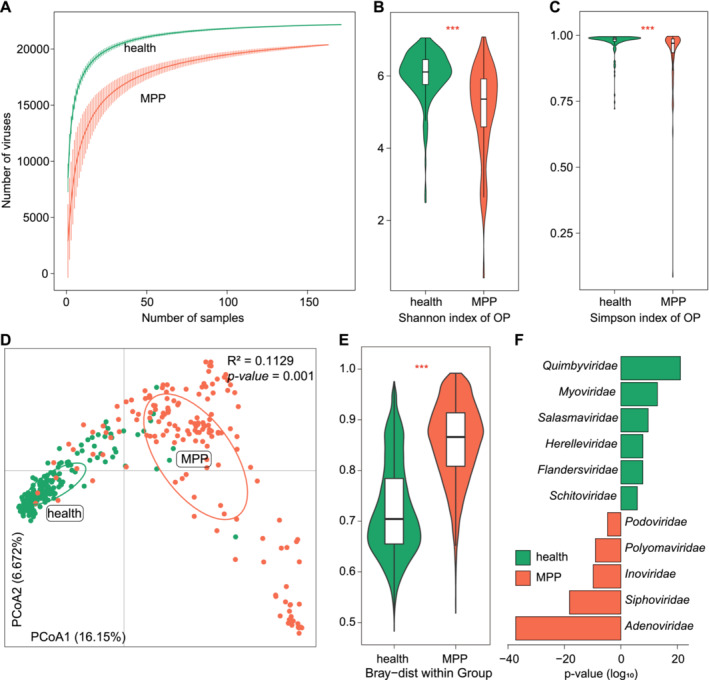
Comparison analysis of oropharynx virome between healthy controls and children with pneumonia. (A) The sample‐based rarefaction curve showing the number of observed viruses within healthy controls and patients. (B,C) The Shannon index and Simpson index of healthy controls and patients. Statistical significance is estimated by Wilcoxon rank‐sum test between two groups. *p < 0.001. (D) Principal coordinate analysis (PCoA) of the Bray–Curtis distance based on the vOTU‐level abundance data. Samples are plotted against the first two principal coordinate axes (PC1 and PC2), and the ratio of variance contributed by these two PCs is shown. The 80% confidence ellipsoids are outlined for each group. Two numbers in the upper right of the figure represent the proportion of explained variation (*R* ^2^) and statistical significance estimated by Adonis test, respectively. (E) The inter‐sample Bray–Curtis distance in healthy controls and patient groups. (F) Statistical difference in viral family‐level composition between healthy controls and patients. The x‐axis is the −log10 transformed *p* values from Wilcoxon rank‐sum tests (HC‐enriched families along the positive x‐axis and PP‐enriched families along the negative x‐axis).

To explore the alteration of virome diversity in the patient's oropharynx, viral richness and evenness of each metagenome were examined using the number of observed vOTUs (estimation for viral richness), Shannon diversity index, and Simpson diversity index. Compared with healthy controls (HC), the pneumonia patients (PP) showed a significant decrease in viral richness and evenness (Wilcoxon rank‐sum test *p* < 0.001; Figure 2A–C). Then, we estimated the effect size of the disease status on overall oropharynx viral composition using principal coordinate analysis (PCoA) and permutational multivariate analysis of variance (PERMANOVA). PCoA of the vOTU‐level profiles clearly separated the healthy controls and pneumonia patients (Figure 2D), and PERMANOVA showed that the disease status explained 11.3% of the variance in oropharynx viral community composition (*adonis p* < 0.001 after adjusting for subjects' sex, age, and other phenotypes). Similarly, analysis of inter‐sample variability also revealed that the viral compositional variability of patients was significantly increased than that of healthy controls (Wilcoxon rank‐sum test *p* < 0.001; Figure 2E). These findings suggested a remarkable difference in oropharynx virome between healthy controls and patients with pneumonia.

Comparison analysis of the family‐level viral composition identified 11 families with significant differences between healthy controls and pneumonia patients (Wilcoxon rank‐sum test with Benjamini‐Hochberg correction *q* < 0.01; Table S4). The HC‐enriched families included Quimbyviridae, Myoviridae, Salasmaviridae, Herelleviridae, Flandersviridae, and Schitoviridae, whereas the PP‐enriched families contained Adenoviridae, Siphoviridae, Inoviridae, Polyomaviridae, and Podoviridae (Figure 2F).

### 3.3 Viral Markers Associated With Pneumonia in Oropharynx

**FIGURE 3:**
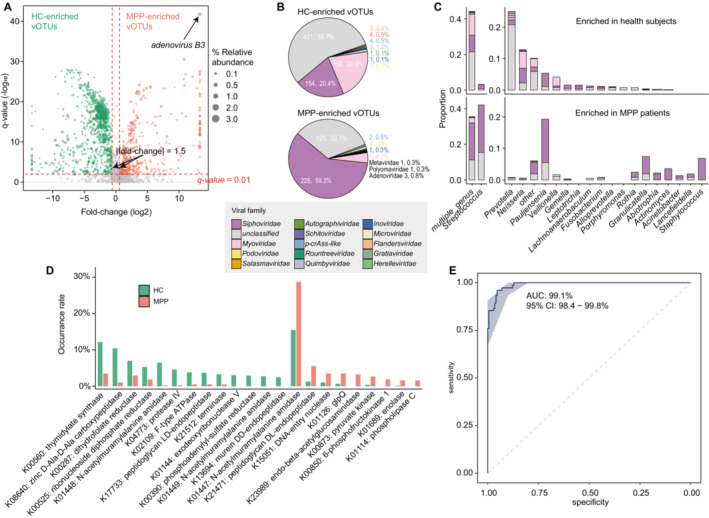
Identification and features of pneumonia‐associated oropharynx viruses. (A) Volcano plot showing significant vOTUs in oropharynx between healthy controls and children with pneumonia. The x‐axis is log2 transformed fold changes in mean relative abundances of vOTUs between two groups (HC‐enriched vOTUs along the negative x‐axis and PP‐enriched vOTUs along the positive x‐axis). The y‐axis is the –log10 transformed *q* values from Wilcoxon rank‐sum tests following Benjamini–Hochberg correction. The size of the point represents the mean relative abundance of vOTU in healthy or patient groups. (B) Family‐level annotation of the HC‐ and PP‐enriched vOTUs. (C) Distribution of prokaryotic hosts of the HC‐ and PP‐enriched vOTUs. The prokaryotic hosts are grouped at the genus level, and the viruses are colored based on their family‐level annotation. (D) Bar plot showing significant functional orthologs (KOs) between the HC‐ and PP‐enriched vOTUs. The occurrence rate of a KO in each group refers to the number of vOTUs with this KO divided by total number of vOTUs. Statistical significance is estimated by Fisher's exact test between two groups, and KOs with *p* values < 0.01 are positive and plotted. (E) Receiver operating characteristic (ROC) analysis showing the performance of the random forest model for classifying healthy controls and patients based on pneumonia‐associated oropharynx viruses. The validation is implemented using 10 repeats of 10‐fold cross‐validation.

A total of 1137 core vOTUs (with average relative abundance > 0.01% across all oropharynx samples) were identified as viral markers associated with pneumonia using Wilcoxon rank‐sum test following Benjamini–Hochberg correction (*q* < 0.01; fold change > 1.5; Figure 3A and Table S5), including 756 HC‐enriched vOTUs and 381 PP‐enriched vOTUs. Taxonomically, the HC‐enriched vOTUs were more often annotated as the members of Myoviridae and as family‐level unclassified viruses, whereas the PP‐enriched vOTUs were more often annotated as the members of Siphoviridae and Podoviridae (Figure 3B). Notably, the HC‐enriched vOTUs included nine Quimbyviridae, four Schitoviridae, and three Flandersviridae viruses, but no member of these families appeared in PP‐enriched vOTUs, whereas five eukaryotic viruses were enriched in patients, including *human adenovirus B3*, *human adenovirus C*, *human adenovirus E4*, *WU polyomavirus*, and one Metaviridae member (Figure 3B). Furthermore, different host preference patterns were observed between HC‐ and PP‐enriched vOTUs (Figure 3C). The hosts of HC‐enriched vOTUs were dominated by members of *Prevotella*, *Neisseria*, *Pauljensenia*, and *Veillonella*, whereas the prevalent hosts of HC‐enriched vOTUs were members of *Streptococcus*, *Pauljensenia*, *Granulicatella*, *Staphylococcus*, and *Actinomyces*. Interestingly, we observed some PP‐enriched vOTUs specifically infecting potential pathogens. For example, 13 Siphoviridae vOTUs were predicted to specifically infect *Staphylococcus aureus* , whereas the predicted host of one Myoviridae vOTU was *Haemophilus parainfluenzae* (Table S5). These viruses may be suitable bacteriophage therapy candidates targeting these organisms.

Given that viral auxiliary metabolic genes (AMGs) potentially affect host metabolism and are associated with the disease risk [33, 34], we predicted 81,587 protein‐coding genes from 1137 viral markers and assigned 3187 of them to 571 metabolism‐associated functional orthologs (i.e., KEGG orthologs [35]) by querying the KEGG database. Comparison analysis was conducted based on the occurrence rates of KOs between HC‐ and PP‐enriched vOTUs generating 22 metabolism‐associated KOs with significant differences (Fisher's exact test *p* < 0.01; Figure 3D and Table S6). The PP‐enriched vOTUs had a higher frequency of nine enzymes including three glycolysis‐associated KOs (K00850, K00873, and K01689), two KOs involving peptidoglycan degradation (K01447 and K21471), and four other KOs (K01114, K01126, K15051, and K23989). By contrast, the HC‐enriched vOTUs had a higher frequency of 13 enzymes including five KOs involving peptidoglycan degradation (K01448, K01449, K08640, K13694, and K17733), three KOs involving nucleotide metabolism (K00560, K01144, and K21512), K00390 (phosphoadenylyl‐sulfate reductase), K00287 (dihydrofolate reductase), and three other KOs (K00525, K02109, and K04773).

Finally, to assess the diagnostic potential of oropharyngeal viral populations in patients with pneumonia, we established a random forest classification model based on the patient‐associated vOTUs to differentiate patients with pneumonia from healthy controls and assessed the performance of the model by measuring the area under the receiver operating characteristic curve (AUC). The classification model showed an outstanding classification efficacy of pneumonia (AUC = 99.1%) and was validated by 10 repeats of 10‐fold cross‐validation (Figure 3D). The finding suggested the possibility of oropharyngeal viruses as predictors of pneumonia.

### 3.4 Mouth–Nose–Lung Transmission of Viral Populations in Patients With Pneumonia

**FIGURE 4:**
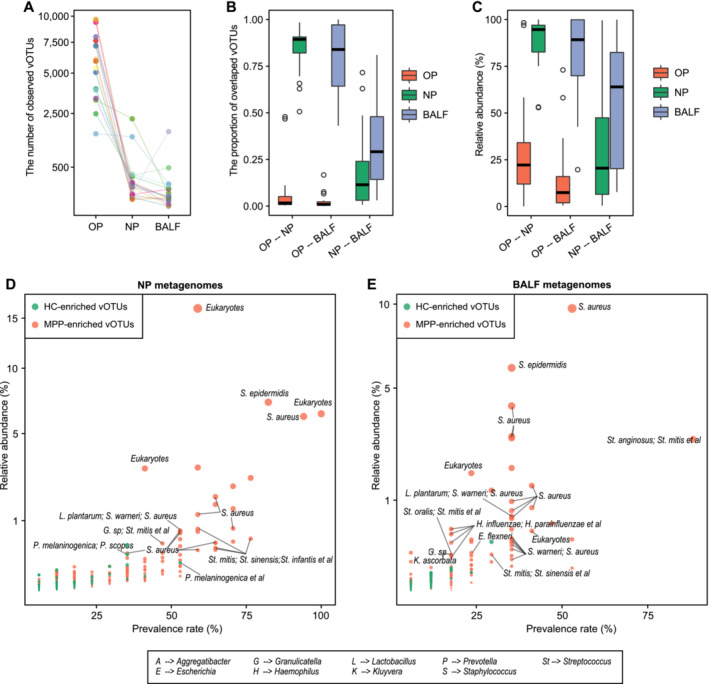
The overlaps of viruses among oropharynx, nasopharynx, and bronchoalveolar lavage fluid of children with pneumonia. (A) Scatter plot showing the total number of vOTUs detected in each sample. Samples from the same individual are connected by a single edge. (B) The distribution of proportions of vOTUs overlapping between two paired samples from any two groups. The proportion is calculated as the number of overlapped vOTUs between two samples from the same individual divided by the total number of vOTUs observed in each sample. (C) The distribution of the total relative abundances of overlapped vOTUs between two paired samples from any two groups. (D) The relative abundances and prevalence rates of pneumonia‐associated oropharynx viruses in nasopharynx samples. Each point represents a vOTU and is labeled by its predicted hosts. The size of points denotes the mean relative abundance of this vOTU in nasopharynx samples. (E) The relative abundances and prevalence rates of pneumonia‐associated oropharynx viruses in bronchoalveolar lavage fluid samples.

To explore the communications among oral, nasal, and lung viral populations, we investigated the distribution of viral populations of 17 OP‐NP‐BALF sample pairs of children with pneumonia. The OP metagenomes showed the largest viral richness compared with the NP and BALF metagenomes (Figure 4A). The majority of vOTUs in NP and BALF metagenomes (average 83.5% and 80.0%) could be detected in OP metagenomes, whereas a few vOTUs in OP metagenomes (< 10%) appeared in NP and BALF metagenomes (Figure 4B). And the vOTUs shared between OP and NP (or BALF) metagenomes accounted for > 80% of the total relative abundance of viral communities in NP or BALF metagenomes (Figure 4C). Besides, there was limited overlap between vOTUs of the NP and BALF metagenomes (< 35%; Figure 4B). These results suggested that lung and nasal viral populations may be dominated by oral viral communities.

We especially focused on the distribution of 1137 pneumonia‐associated viral markers (identified in OP metagenomes) in NP and BALF metagenomes. In NP metagenomes, 646 of these viral markers were detectable, including 326 control‐enriched vOTUs and 320 patient‐enriched vOTUs. Compared with the control‐enriched vOTUs, the patient‐enriched vOTUs showed higher relative abundances and prevalence rates, such as *WU polyomavirus*, *human adenovirus*, phages infecting *S. aureus* , *Staphylococcus epidermidis* , and *Streptococcus mitis* (Figure 4D). Likewise, in BALF metagenomes, compared with the 295 detectable control‐enriched vOTUs, the 266 patient‐enriched vOTUs exhibited higher abundance levels and prevalence rates, such as *human adenovirus*, phages infecting members of *S. aureus* , *S. mitis* , *H. influenzae* , and *H. parainfluenzae* (Figure 4E). These results suggested frequent communications of viruses enriched in pneumonia patients among the oropharynx, nasopharynx, and lung.

### 3.5 Metagenomic Analysis as an Auxiliary Diagnostic Method for Pathogen Detection

The previous study had used targeted polymerase chain reaction (PCR)‐based tests to detect common pathogens in BALF specimens, including two bacteria ( *M. pneumoniae* and *Chlamydia pneumonia*) and five types of viruses (cytomegalovirus, Epstein–Barr virus, human adenovirus, influenza virus, parainfluenza virus, and respiratory syncytial virus) [22]. Based on our virome analysis, we found that two BALF metagenomes (NCBI accession SAMN07758852 and SAMN07758861) exhibited an abnormally high rate of reads mapping the respiratory tract viral genome catalog (Table 1), whereas both of them tested negative for viruses by targeted PCR‐based tests [22].

**TABLE 1:** Characteristics of study population and metagenomic sequencing data.

		Metagenomic result						qPCR result	
Patient (gender, age)	Pathogen detection	NCBI accession	Sample type	# total clean reads	% clean reads (all viruses)	% clean reads (all prokaryotes)	% clean reads (pathogen)	Sample type	Test result
P12 (female, 4 years)	*Human adenovirus (B3, C)* *Mycoplasma pneumoniae*	SAMN07758852	BALF	537,056	75.06	6.28	74.98 2.55	BALF	Neg Pos
P45 (female, < 1 year)	*Human adenovirus (C)* *M. pneumoniae*	SAMN07758861	BALF	50,172	37.59	2.26	37.57 0.32	BALF	Neg Neg
P56 (male, 1 year)	*WU polyomavirus* *Streptococcus pseudopneumoniae* *Streptococcus pneumoniae* *M. pneumoniae*	SAMN07758828	OP	9,601,936	78.95	19.83	77.41 0.44 0.18 < 0.01	BALF	NA NA NA Neg
P59 (male, 2 years)	*Human adenovirus (B3)* *WU polyomavirus* *S. pneumoniae* *M. pneumoniae* *S. pseudopneumoniae*	SAMN07758889	NP	19,640	63.86	13.41	54.05 9.38 0.12 0.07 0.06	BALF	Pos NA NA Pos NA
P29 (female, 4 years)	*Human adenovirus (B3, C)* *S. pneumoniae* *M. pneumoniae*	SAMN07758881	NP	90,410	77.34	15.56	77.06 0.04 0.01	BALF	Neg NA Pos
P64 (female, 5 years)	*Human adenovirus (B3)* *WU polyomavirus* *S. pneumoniae* *M. pneumoniae*	SAMN07758892	NP	103,946	77.96	2.44	77.60 0.29 0.03 0.02	BALF	Neg NA NA Pos

#### 3.5.1 BALF Metagenome 1 (SAMN07758852)

Metagenomic analysis revealed that human adenovirus accounted for 74.98% of total clean reads, whereas only 6.28% of reads were captured by prokaryotes, especially *M. pneumoniae* (2.55%), suggesting human adenovirus serving as a major pathogen for this patient. Only *M. pneumoniae* tested positive by the qPCR‐based test on this BALF specimen.

#### 3.5.2 BALF Metagenome 2 (SAMN07758861)

Metagenomic analysis showed that viral populations and prokaryotes captured 37.59% and 2.26% clean reads, respectively. Human adenovirus accounted for 37.57% of clean reads, which was remarkably higher than that of other detected microbes. The qPCR‐based test was not able to detect any pathogen.

Additionally, we noted that one OP (SAMN07758828) and three NP (SAMN07758889, SAMN07758881, and SAMN07758892) metagenomes also showed a high rate of clean reads mapping the viral genome catalog (Table 1). Unfortunately, due to the lack of available BALF metagenomes corresponding to the above four metagenomes [22], we could not explore their lung viral loads.

#### 3.5.3 OP Metagenome 1 (SAMN07758828)

Metagenome analysis showed that viral populations captured 78.95% of total clean reads, especially *polyomavirus* (accounting for 77.41%), whereas 19.83% of clean reads were recruited by prokaryotes. Notably, some prokaryotic pathogens recruited a very low proportion of clean reads, such as *Streptococcus pseudopneumoniae* (0.44%), *S. pneumoniae* (0.18%), and *M. pneumoniae* (< 0.01%). The PCR‐based test was not able to detect any pathogen on the BALF of the same patient.

#### 3.5.4 NP Metagenome 1 (SAMN07758889)

Metagenomic analysis revealed that viral populations captured 63.9% of total clean reads, especially human adenovirus and *polyomavirus* (accounting for 54.05% and 9.38%, respectively), whereas 13.4% of clean reads were recruited by prokaryotes. Some prokaryotic pathogens recruited a very low proportion of clean reads, such as *S. pneumoniae* (0.11%) and *M. pneumoniae* (0.07%). Human adenovirus and *M. pneumoniae* were tested positive by the PCR‐based test on the BALF of the same patient.

#### 3.5.5 NP Metagenomes 2 and 3 (SAMN07758881 and SAMN07758892)

Both NP metagenomes showed that a majority of clean reads (77.34% and 77.96%) were recruited by human adenovirus. *M. pneumoniae* recruited a very low proportion of clean reads in the two metagenomes (0.01% and 0.02%). The PCR‐based test only detected *M. pneumoniae* on BALF of the same patient.

These results indicated that metagenomic analysis could serve as an effective diagnostic tool for pathogen detection and make up for the limitations of targeted PCR‐based tests. Besides, metagenomic analysis for OP and NP swabs may provide a timely diagnosis of pathogen detection.

## 4 Discussion

Herein, we reanalyzed the public respiratory tract metagenomes sequenced from the OP swab, NP swab, and BALF specimens of 76 child patients with pneumonia and the OP specimens of 171 healthy children. The de novo assembly and the combined homology‐ and feature‐based methods were applied in this study for viral identification to comprehensively characterize respiratory tract virome composition in whole‐genome DNA sequencing samples of children with pneumonia. Identification of respiratory tract virome may facilitate further mechanistic studies of children's pneumonia and other related diseases.

We developed a comprehensive viral genome database that encompasses both URT and LRT viruses. Notably, unlike previous studies that focused on common eukaryotic viruses [35, 36, 37], our database not only provided genome sequences of eukaryotic viruses but also included a substantial number of prokaryotic viral genomes. Many of these prokaryotic viruses as bacteriophages were predicted to infect respiratory pathogens such as *H. influenzae* , *S. pneumoniae* , *S. pseudopneumoniae* , *S. aureus* , and others. Given the increasing prevalence of antibiotic‐resistant strains due to widespread antibiotic usage, there has been a renewed interest in bacteriophage therapy among researchers [38]. The prokaryotic viruses identified in our study could serve as a valuable resource for future consideration in bacteriophage interventions targeting respiratory infections. Based on this genome collection, we profiled the viral community composition of all respiratory tract metagenomes and found a remarkable difference in viral composition at the family level among OP, NP, and BALF metagenomes. Specifically, the higher proportions of Adenoviridae and Polyomaviridae in NP and BALF samples compared to OP samples can be explained by their association with pneumonia‐causing viruses [39, 40]. Furthermore, we observed frequent viral transmission between OP, NP, and BALF samples, particularly for viruses enriched in the OP samples of patients with pneumonia, which also displayed a high relative abundance in NP and BALF samples. These findings highlighted the dynamic interplay between the URT and LRT viromes and hinted the potential of OP samples for monitoring and evaluating the lung micro‐ecosystem.

We also found a remarkable difference in oropharynx virome diversity and composition between healthy controls and patients with pneumonia. The control‐enriched families included Quimbyviridae, Myoviridae, Salasmaviridae, Herelleviridae, Flandersviridae, and Schitoviridae, whereas the patient‐enriched families contained Adenoviridae, Siphoviridae, Inoviridae, Polyomaviridae, and Podoviridae. Similarly, the gut virome of patients with COVID‐19 has shown the presence of Herelleviridae, Virgaviridae, crAss‐like phage, Inoviridae, Microviridae, Myoviridae, Podoviridae, and Siphoviridae family of viruses [41]. Quimbioviridae is described viral family with highly abundant and widely prevalent in the human gut and is considered as an obligate lytic phage, and some Quimbioviridae phages have also been reported to produce a wide diversity of retroelements (DGRs), producing highly variable target genes nested in defense‐related genes [42]. The relative abundance of Quimbioviridae was found to increase in patients with polycystic ovary syndrome, and Bacteroidaceae were the mostly bacterial hosts of members of the Quimbioviridae [43].

Our results then showed a total of 1137 vOTUs that exhibited a significant difference between oropharynx samples obtained from children with pneumonia and those from healthy controls. The pneumonia‐enriched vOTUs mainly included *polyomavirus*, *human adenovirus*, and phages infecting the *Staphylococcus*, *Streptococcus*, *Granulicatella*, and *Actinomyces*. These genera encompass a variety of respiratory pathogens, such as *S. pneumoniae* and *S. aureus* . Numerous studies have demonstrated that the lungs of healthy individuals harbor microbiota, with the six predominant bacterial species identified as members of *Prevotella*, *Streptococcus*, *Veillonella*, *Fusobacterium*, *Porphyromonas*, and *Neisseria* [44]. These bacteria are also the most abundant in the oral cavity, suggesting that viruses infecting them may frequently exchange between the oral cavity and the lungs. Consequently, we attempted to assess the diagnostic potential of pneumonia‐associated oropharyngeal viruses in pneumonia patients. The results demonstrated outstanding classification efficacy for pneumonia (AUC = 99.1%). This finding suggests the possibility of oropharyngeal viruses as predictors of pneumonia, which could be advantageous for more convenient detection of pulmonary infections in practical applications.

Notably, virome analysis exhibited that six child patients had a severe infection of *human adenovirus* or *WU polyomavirus* and five of them may go undiagnosed in targeted PCR‐based test results. Human adenovirus is frequently detected in respiratory samples of children with respiratory infections and often causes mild to moderate symptoms [45]. In our study, a BALF sample exhibited a high proportion of sequences from both human adenovirus and *M. pneumoniae* . Specifically, human adenovirus accounted for 74.98% of the microbial sequences in the BALF sample, which was significantly higher than the proportion of *M. pneumoniae* (2.55%). The patient was experiencing a mixed viral‐bacterial infection, with human adenovirus potentially playing a primary triggering factor. Interestingly, despite the original authors mentioning that they used a targeted PCR‐based test for the detection of *M. pneumoniae* and human adenovirus, the sample only reported a positive result for *M. pneumoniae* and did not report a positive result for human adenovirus [22]. One possible explanation is that the strain heterogeneity of human adenovirus led to a decrease in PCR primer sensitivity. This suggests that relying solely on targeted PCR‐based tests to determine pathogen positivity may be insufficient. Combining NGS analysis with quantitative results can help improve pathogen identification. On the other hand, since its discovery in 2007, *WU polyomavirus* has been detected in 2%–5% of patients with respiratory disease [46, 47, 48], and it has been reported to have independent pathogenicity [40]. In our study, there was a finding in which most microbial sequences in an oral sample were attributed to WU polyomavirus. The oral microbial composition typically consists primarily of prokaryotes [26]. The presence of WU polyomavirus in such abundance suggested that it may be responsible for the severe clinical symptoms observed. However, the original authors did not perform targeted PCR‐based tests for WU polyomavirus in the samples [22]. Overall, the virome analysis not only aided in the identification of pathogens that were overlooked by targeted PCR‐based tests but also emphasized the significance of quantifying the abundance of each pathogen in a sample for targeted treatment.

It should be noted that this paper also has several limitations. Firstly, the most shortcoming is that the study is based on metagenomic DNA sequencing samples, which does not allow for the investigation of the RNA virome. Secondly, due to the large number of unknown viruses identified in PP‐associated viruses, research on their pathogenic mechanisms is greatly restricted. Thirdly, as we were unable to collect BALF samples from healthy individuals, we could not conduct a case–control study on the pulmonary virome.

## 5 Conclusions

This work characterized the respiratory tract virome of children with pneumonia, showed frequent transmission of potentially pathogenic viruses in the respiratory tract, and highlighted the effective capacity of virome analysis serving as an auxiliary diagnostic method for pathogen detection.

## Author Contributions

**Song Cui:** conceptualization, investigation, writing – original draft. **Ruochun Guo:** conceptualization, data curation, formal analysis, methodology, software. **Changming Chen:** conceptualization, validation, visualization, writing – original draft. **Yue Zhang:** data curation, formal analysis, investigation, methodology, software, visualization. **Jinxin Meng:** data curation, formal analysis, writing – original draft. **Lanxin Liu:** resources, supervision, validation, writing – review and editing. **Yanxia Li:** data curation, formal analysis, writing – review and editing. **Zhijie Kang:** resources, validation, visualization, writing – review and editing. **Shenghui Li:** conceptualization, investigation, methodology, writing – original draft, writing – review and editing. **Qiulong Yan:** conceptualization, funding acquisition, project administration, supervision, writing – review and editing. **Yufang Ma:** conceptualization, project administration, resources, supervision, writing – review and editing.

## Conflicts of Interest

The authors declare no competing interests.

## Supporting information


**Table S1** Metadata and data information of each metagenomic sample.
**Table S2.** Genome quality assessment, taxonomy annotation, and host assignment of 2402 vOTUs recovered from 334 respiratory tract metagenomes.
**Table S3.** Clustering information, taxonomy annotation, and host assignment of a combined viral genome catalog including 22,860 vOTUs.
**Table S4.** Comparison analysis of the viral family‐level composition in oropharynx between healthy controls (HC) and children with pneumonia (PP).
**Table S5.** Detail information of 1137 pneumonia‐associated vOTUs in oropharynx.
**Table S6.** Comparison analysis of the occurrence rates of functional orthologs between HC‐ and PP‐enriched vOTUs in oropharynx.

## Data Availability

All data needed to evaluate the conclusions in the paper are present in the paper and/or the Supporting Information.

## References

[1] B. Dadonaite , and M. Roser , “Pneumonia,” (2019), https://ourworldindataorg/pneumonia.

[2] S. Mahtab , D. M. Blau , Z. J. Madewell , et al., “Post‐mortem Investigation of Deaths due to Pneumonia in Children Aged 1–59 Months in Sub‐Saharan Africa and South Asia From 2016 to 2022: An Observational Study,” The Lancet Child & Adolescent Health 8, no. 3 (2024): 201–213. 38281495 10.1016/S2352-4642(23)00328-0 PMC10864189

[3] M. Shehu , M. M. Ihekaike , and H. Shehu , “Comparing the Incidence of Pneumonia in Children Seen at a Nigerian Teaching Hospital Before and During the COVID‐19 Pandemic,” Nigerian Journal of Paediatrics 49, no. 2 (2022): 131–135.

[4] WHO , “Pneumonia in Children,” (2022), https://wwwwhoint/news‐room/fact‐sheets/detail/pneumonia.

[5] C. Thibeault , N. Suttorp , and B. Opitz , “The Microbiota in Pneumonia: From Protection to Predisposition,” Science Translational Medicine 13, no. 576 (2021): eaba0501. 33441423 10.1126/scitranslmed.aba0501

[6] S. A. Whiteside , J. E. McGinniss , and R. G. Collman , “The Lung Microbiome: Progress and Promise,” The Journal of Clinical Investigation 131, no. 15 (2021): e150473. 34338230 10.1172/JCI150473 PMC8321564

[7] B. G. Wu , I. Sulaiman , J.‐C. J. Tsay , et al., “Episodic Aspiration With Oral Commensals Induces a MyD88‐Dependent, Pulmonary T‐Helper Cell Type 17 Response That Mitigates Susceptibility to *Streptococcus pneumoniae* ,” American Journal of Respiratory and Critical Care Medicine 203, no. 9 (2021): 1099–1111. 33166473 10.1164/rccm.202005-1596OC PMC8314894

[8] R. Li , J. Li , and X. Zhou , “Lung Microbiome: New Insights Into the Pathogenesis of Respiratory Diseases,” Signal Transduction and Targeted Therapy 9, no. 1 (2024): 19. 38228603 10.1038/s41392-023-01722-y PMC10791971

[9] H. J. Zar , R. MacGinty , L. Workman , et al., “ *Klebsiella pneumoniae* Lower Respiratory Tract Infection in a South African Birth Cohort: A Longitudinal Study,” International Journal of Infectious Diseases 121 (2022): 31–38. 35472523 10.1016/j.ijid.2022.04.043 PMC9174060

[10] S. Bianchini , E. Silvestri , A. Argentiero , V. Fainardi , G. Pisi , and S. Esposito , “Role of Respiratory Syncytial Virus in Pediatric Pneumonia,” Microorganisms 8, no. 12 (2020): 2048. 33371276 10.3390/microorganisms8122048 PMC7766387

[11] X. Zhao , Y. Meng , D. Li , et al., “Retrospective Study of Clinical Characteristics and Viral Etiologies of Patients With Viral Pneumonia in Beijing,” Pulmonary Circulation 11, no. 2 (2021): 20458940211011027. 34221349 10.1177/20458940211011027 PMC8221751

[12] M. T. Pratt , T. Abdalla , P. C. Richmond , et al., “Prevalence of Respiratory Viruses in Community‐Acquired Pneumonia in Children: A Systematic Review and Meta‐Analysis,” The Lancet Child & Adolescent Health 6, no. 8 (2022): 555–570. 35636455 10.1016/S2352-4642(22)00092-X

[13] A. C. Gregory , O. Zablocki , A. A. Zayed , A. Howell , B. Bolduc , and M. B. Sullivan , “The Gut Virome Database Reveals Age‐Dependent Patterns of Virome Diversity in the Human Gut,” Cell Host & Microbe 28, no. 5 (2020): 724–740.e8. 32841606 10.1016/j.chom.2020.08.003 PMC7443397

[14] S. Nayfach , D. Paez‐Espino , L. Call , et al., “Metagenomic Compendium of 189,680 DNA Viruses From the Human GHut Microbiome,” Nature Microbiology 6, no. 7 (2021): 960–970. 10.1038/s41564-021-00928-6 PMC8241571 34168315

[15] S. Li , R. Guo , Y. Zhang , et al., “A Catalog of 48,425 Nonredundant Viruses From Oral Metagenomes Expands the Horizon of the Human Oral Virome,” iScience 25, no. 6 (2022): 104418. 35663034 10.1016/j.isci.2022.104418 PMC9160773

[16] S. Saheb Kashaf , D. M. Proctor , C. Deming , et al., “Integrating Cultivation and Metagenomics for a Multi‐kingdom View of Skin Microbiome Diversity and Functions,” Nature Microbiology 7, no. 1 (2022): 169–179. 10.1038/s41564-021-01011-w PMC8732310 34952941

[17] L. Huang , R. Guo , S. Li , et al., “A Multi‐kingdom Collection of 33,804 Reference Genomes for the Human Vaginal Microbiome,” Nature Microbiology (2024), 10.1038/s41564-024-01751-5. PMC11306104 38907008

[18] B. N. Porto , “Insights Into the Role of the Lung Virome During Respiratory Viral Infections,” Frontiers in Immunology 13 (2022): 885341. 35572506 10.3389/fimmu.2022.885341 PMC9091589

[19] A. Singanayagam , P. V. Joshi , P. Mallia , and S. L. Johnston , “Viruses Exacerbating Chronic Pulmonary Disease: The Role of Immune Modulation,” BMC Medicine 10 (2012): 27. 22420941 10.1186/1741-7015-10-27 PMC3353868

[20] D. Willner , M. Furlan , M. Haynes , et al., “Metagenomic Analysis of Respiratory Tract DNA Viral Communities in Cystic Fibrosis and Non‐cystic Fibrosis Individuals,” PLoS ONE 4, no. 10 (2009): e7370. 19816605 10.1371/journal.pone.0007370 PMC2756586

[21] X. Chen , Y. Kang , J. Luo , et al., “Next‐Generation Sequencing Reveals the Progression of COVID‐19,” Frontiers in Cellular and Infection Microbiology 11 (2021): 632490. 33777844 10.3389/fcimb.2021.632490 PMC7991797

[22] W. Dai , H. Wang , Q. Zhou , et al., “An Integrated Respiratory Microbial Gene Catalogue to Better Understand the Microbial Aetiology of Mycoplasma Pneumoniae Pneumonia,” GigaScience 8, no. 8 (2019): giz093. 31367746 10.1093/gigascience/giz093 PMC6669060

[23] S. Chen , Y. Zhou , Y. Chen , and J. Gu , “Fastp: An Ultra‐Fast all‐In‐One FASTQ Preprocessor,” Bioinformatics 34, no. 17 (2018): i884–i890. 30423086 10.1093/bioinformatics/bty560 PMC6129281

[24] B. Langmead and S. L. Salzberg , “Fast Gapped‐Read Alignment With Bowtie 2,” Nature Methods 9, no. 4 (2012): 357–359. 22388286 10.1038/nmeth.1923 PMC3322381

[25] D. Li , C. M. Liu , R. Luo , K. Sadakane , and T. W. Lam , “MEGAHIT: An Ultra‐Fast Single‐Node Solution for Large and Complex Metagenomics Assembly via Succinct de Bruijn Graph,” Bioinformatics 31, no. 10 (2015): 1674–1676. 25609793 10.1093/bioinformatics/btv033

[26] J. Zhu , L. Tian , P. Chen , et al., “Over 50,000 Metagenomically Assembled Draft Genomes for the Human Oral Microbiome Reveal New Taxa,” Genomics, Proteomics & Bioinformatics 20 (2022): 246–259. 10.1016/j.gpb.2021.05.001 PMC9684161 34492339

[27] S. Nayfach , A. P. Camargo , F. Schulz , E. Eloe‐Fadrosh , S. Roux , and N. C. Kyrpides , “CheckV Assesses the Quality and Completeness of Metagenome‐Assembled Viral Genomes,” Nature Biotechnology 39 (2021): 578–585. 10.1038/s41587-020-00774-7 PMC8116208 33349699

[28] J. Ren , K. Song , C. Deng , et al., “Identifying Viruses From Metagenomic Data Using Deep Learning,” Quantitative Biology 8, no. 1 (2020): 64–77. 34084563 10.1007/s40484-019-0187-4 PMC8172088

[29] K. Kieft , Z. Zhou , and K. Anantharaman , “VIBRANT: Automated Recovery, Annotation and Curation of Microbial Viruses, and Evaluation of Viral Community Function From Genomic Sequences,” Microbiome 8, no. 1 (2020): 90. 32522236 10.1186/s40168-020-00867-0 PMC7288430

[30] D. Hyatt , G.‐L. Chen , P. F. LoCascio , M. L. Land , F. W. Larimer , and L. J. Hauser , “Prodigal: Prokaryotic Gene Recognition and Translation Initiation Site Identification,” BMC Bioinformatics 11, no. 1 (2010): 1–11. 20211023 10.1186/1471-2105-11-119 PMC2848648

[31] B. Buchfink , C. Xie , and D. H. Huson , “Fast and Sensitive Protein Alignment Using DIAMOND,” Nature Methods 12, no. 1 (2015): 59–60. 25402007 10.1038/nmeth.3176

[32] M. Kanehisa , Y. Sato , M. Kawashima , M. Furumichi , and M. Tanabe , “KEGG as a Reference Resource for Gene and Protein Annotation,” Nucleic Acids Research 44, no. D1 (2016): D457–D462. 26476454 10.1093/nar/gkv1070 PMC4702792

[33] L. R. Thompson , Q. Zeng , L. Kelly , et al., “Phage Auxiliary Metabolic Genes and the Redirection of Cyanobacterial Host Carbon Metabolism,” Proceedings of the National Academy of Sciences of the United States of America 108, no. 39 (2011): E757–E764. 21844365 10.1073/pnas.1102164108 PMC3182688

[34] M. R. Mangalea , D. Paez‐Espino , K. Kieft , et al., “Individuals at Risk for Rheumatoid Arthritis Harbor Differential Intestinal Bacteriophage Communities With Distinct Metabolic Potential,” Cell Host & Microbe 29, no. 5 (2021): 726–739.e5. 33957082 10.1016/j.chom.2021.03.020 PMC8186507

[35] A. L. Van Rijn , S. Van Boheemen , I. Sidorov , et al., “The Respiratory Virome and Exacerbations in Patients With Chronic Obstructive Pulmonary Disease,” PLoS ONE 14, no. 10 (2019): e0223952. 31647831 10.1371/journal.pone.0223952 PMC6812800

[36] S. V. Rajagopala , N. G. Bakhoum , S. B. Pakala , et al., “Metatranscriptomics to Characterize Respiratory Virome, Microbiome, and Host Response Directly From Clinical Samples,” Cell Reports Methods 1, no. 6 (2021): 100091. 34790908 10.1016/j.crmeth.2021.100091 PMC8594859

[37] A. E. Ogunbayo , M. T. Mogotsi , H. Sondlane , K. R. Nkwadipo , S. Sabiu , and M. M. Nyaga , “Metagenomic Analysis of Respiratory RNA Virome of Children With and Without Severe Acute Respiratory Infection From the Free State, South Africa During COVID‐19 Pandemic Reveals Higher Diversity and Abundance in Summer Compared With Winter Period,” Viruses 14, no. 11 (2022): 2516. 36423125 10.3390/v14112516 PMC9692838

[38] G. F. Hatfull , R. M. Dedrick , and R. T. Schooley , “Phage Therapy for Antibiotic‐Resistant Bacterial Infections,” Annual Review of Medicine 73 (2022): 197–211. 10.1146/annurev-med-080219-122208 34428079

[39] Y. L‐h , C. Wang , W. T‐l , H. Wang , F.‐l. Ma , and L.‐s. Zheng , “Human Adenovirus Among Hospitalized Children With Respiratory Tract Infections in Beijing, China, 2017–2018,” Virology Journal 16, no. 1 (2019): 1–8. 31196108 10.1186/s12985-019-1185-x PMC6567909

[40] K. Uda , C. Koyama‐Wakai , K. Shoji , et al., “WU Polyomavirus Detected in Children With Severe Respiratory Failure,” Journal of Clinical Virology 107 (2018): 25–28. 30114678 10.1016/j.jcv.2018.08.003 PMC7106500

[41] J. Cao , C. Wang , Y. Zhang , et al., “Integrated Gut Virome and Bacteriome Dynamics in COVID‐19 Patients,” Gut Microbes 13, no. 1 (2021): 1–21. 10.1080/19490976.2021.1887722 PMC7946006 33678150

[42] S. Benler , N. Yutin , D. Antipov , et al., “Thousands of Previously Unknown Phages Discovered in Whole‐Community Human Gut Metagenomes,” Microbiome 9, no. 1 (2021): 78. 33781338 10.1186/s40168-021-01017-w PMC8008677

[43] L. Huang , X. Wu , S. Guo , et al., “Metagenomic‐Based Characterization of the Gut Virome in Patients With Polycystic Ovary Syndrome,” Frontiers in Microbiology 13 (2022): 951782. 36051758 10.3389/fmicb.2022.951782 PMC9424824

[44] T. P. Wypych , L. C. Wickramasinghe , and B. J. Marsland , “The Influence of the Microbiome on Respiratory Health,” Nature Immunology 20, no. 10 (2019): 1279–1290. 31501577 10.1038/s41590-019-0451-9

[45] T. Lion , “Adenovirus Infections in Immunocompetent and Immunocompromised Patients,” Clinical Microbiology Reviews 27, no. 3 (2014): 441–462. 24982316 10.1128/CMR.00116-13 PMC4135893

[46] X. Y. Cai , Q. Wang , G. Y. Lin , et al., “Respiratory Virus Infections Among Children in South China,” Journal of Medical Virology 86, no. 7 (2014): 1249–1255. 24619492 10.1002/jmv.23931

[47] B.‐M. Le , L. M. Demertzis , G. Wu , et al., “Clinical and Epidemiologic Characterization of WU Polyomavirus Infection, St. Louis, Missouri,” Emerging Infectious Diseases 13, no. 12 (2007): 1936–1938. 18258052 10.3201/eid1312.070977 PMC2876771

[48] K. Subramoney , O. Hellferscee , M. Pretorius , et al., “Human Bocavirus, Coronavirus, and Polyomavirus Detected Among Patients Hospitalised With Severe Acute Respiratory Illness in South Africa, 2012 to 2013,” Health Science Reports 1, no. 8 (2018): e59. 30623094 10.1002/hsr2.59 PMC6266378

